# *Spirulina platensis* Immobilized Alginate Beads for Removal of Pb(II) from Aqueous Solutions

**DOI:** 10.3390/ijerph20021106

**Published:** 2023-01-08

**Authors:** Oyunbileg Purev, Chulhyun Park, Hyunsoo Kim, Eunji Myung, Nagchoul Choi, Kanghee Cho

**Affiliations:** 1 Department of Energy and Resource Engineering, Chosun University, Gwangju 61452, Republic of Korea; 2 Green-Bio Research Facility Center, Seoul National University, Seoul 25354, Republic of Korea; 3 Research Institute of Agriculture and Life Sciences, Seoul National University, Seoul 08826, Republic of Korea

**Keywords:** *Spirulina platensis*, alginate, immobilization, Pb(Ⅱ) removal, N-containing functional groups

## Abstract

Microalgae contain a diversity of functional groups that can be used as environmental adsorbents. *Spirulina platensis* is a blue-green microalga that comprises protein-N, which is advantageous for use in nitrogen-containing biomass as adsorbents. This study aimed to enhance the adsorption properties of alginate hydrogels by employing *Spirulina platensis*. *Spirulina platensis* was immobilized on sodium alginate (S.P@Ca-SA) via crosslinking. The results of field-emission scanning electron microscopy, Fourier-transform infrared, and X-ray photoelectron spectroscopy analyses of the N-containing functional groups indicated that *Spirulina platensis* was successfully immobilized on the alginate matrix. We evaluated the effects of pH, concentration, and contact time on Pb(II) adsorption by S.P@Ca-SA. The results demonstrated that S.P@Ca-SA could effectively eliminate Pb(II) at pH 5, reaching equilibrium within 6 h, and the maximum Pb(II) sorption capacity of S.P@Ca-SA was 87.9 mg/g. Our results indicated that S.P@Ca-SA fits well with the pseudo-second-order and Freundlich models. Compared with *Spirulina platensis* and blank alginate beads, S.P@Ca-SA exhibited an enhanced Pb(II) adsorption efficiency. The correlation implies that the amino groups act as adsorption sites facilitating the elimination of Pb(II).

## 1. Introduction

In the last few decades, industrialization and urbanization have triggered serious environmental issues, with irreparable damage to the ecological environment and human health. Wastewater contaminated with heavy metals is generated in large quantities from natural resources, such as tailings, and diverse industries, including electroplating, smelting, battery and paint manufacture, coating, and mining [1,2,3]. For instance, in mining industries, mineral processing discharges a huge amount of wastewater comprising hazardous metal ions annually. Of the numerous heavy metals employed industrially, Pb(II) is highly toxic and has adverse effects that are injurious to human and animal health because it is bio-accumulative and non-biodegradable. It enters the body predominantly through the skin and food, leading to brain disorders, anemia, kidney disease, and other diseases [4]. Owing to its high toxicity and irreversible damage, South Korea recommends that the concentration of Pb(II) in industrial wastewater should not exceed 0.5 mg/L.

To date, many technologies involving physicochemical and biological approaches, including adsorption, chemical precipitation, and ion exchange, have been adopted to remove heavy metal ions from wastewater [5,6,7,8,9,10,11]. Among these, adsorption has been established as effective in eliminating heavy metal ions, and studies on diverse adsorbents are gaining attention [12,13]. Cost-effective adsorbent-based adsorption techniques have become promising treatment approaches in recent years because of their economic viability and environmental friendliness [14,15]. Currently, activated carbons are the most commonly available adsorbents for heavy metal elimination in wastewater treatment due to their highly porous structure, high specific surface area, and flexibility for surface modification [16]. However, coal-based activated carbons are expensive, and the subsequent pore blockage after heavy metal adsorption degrades their porous structure. Hence, the development of a cost-effective adsorbent that is not only renewable but also has high adsorption capability is the need of the hour.

A variety of agricultural and industrial wastes have been employed for the preparation of low-cost adsorbents. Adsorbents based on agricultural waste are considered cost-effective because of their diverse functional groups, including carboxyl, phenolic, and hydroxyl groups, and easy accessibility. However, the drawback of most waste-based adsorbents lies in the complexity of the adsorbent preparation process [17,18]. Additionally, these adsorbents are synthesized through thermal activation and chemical modifications [19,20].

In recent years, microalgae have captivated increasing attention owing to their simple cultivation and high biomass productivity [21,22]. Microalgae are photosynthetic organisms that are naturally abundant in many regions of the world [23]. Given the significance and rich sources of bioactive compounds with physiological effects, microalgae have drawn interest from researchers and food manufacturers because of their high protein and nutritional content. Moreover, they can be adopted as an adsorbent owing to their high biomass productivity [24]. Among the numerous microalgae species, *Spirulina platensis* is a blue-green microalga that comprises predominantly protein (55–70% of the total dry weight) and has a high nutritional value, including provitamin A, vitamins C and E, essential amino acids, and unsaturated fatty acids [25,26]. The key photosynthetic pigment of *Spirulina platensis* is the dense blue phycocyanin [17]. The surface layers of *Spirulina platensis* have multiple functional groups, including –COOH, –PO_4_, –OH, –SO_4_ –NH_2_, and –SH [27,28]. Therefore, the functional groups of *Spirulina platensis* can be employed for the sorption of heavy metals and dyes from aqueous solutions [21,22,23,24]. 

Considering this, microalgae-derived biochar with a high adsorption capacity is extensively exploited for adsorption studies, but its carbon content is lower than that of coal-based carbons [29,30,31,32]. Additionally, microalgae with low density or biochars are relatively limited, which is a challenge during separation after wastewater treatment [33,34]. This study aimed to enhance the properties of alginate hydrogels using microalgae. We selected *Spirulina platensis* as a low-cost adsorbent due to its enriched N-containing functional groups. More adsorption sites can be exposed, and stability can also be enhanced when sodium alginate composites with *Spirulina platensis* are utilized. Sodium alginate was synthesized using an in situ crosslinking process and adopted for *Spirulina platensis* immobilization. Sodium alginate–*Spirulina platensis* can create hydrogels via Ca^2+^ crosslinking, comprising more controllable particle size and plentiful functional groups, including amino, hydroxyl, and carboxyl groups. We evaluated the adsorption of lead from aqueous solutions of sodium alginate–*Spirulina platensis*.

## 2. Materials and Methods

### 2.1. Materials

*Spirulina platensis* powder was purchased from Qingdao Haixingyuan Biotechnology Co. Ltd. (Qingdao, China) The powder was washed thrice with distilled water to eliminate impurities and collected by centrifugation at 5000 rpm for 10 min. The washed powder was then dried overnight at 60 °C. The dried powder was pulverized and sieved using a standard sieve with a 200 mesh size. Sodium alginate (90%, SA) and calcium chloride (99%, CaCl_2_·2H_2_O) were obtained from Duksan Pure Chemicals (Ansan, Republic of Korea) and used to prepare alginate beads via Ca^2+^ crosslinking. Lead nitrate (99%, Pb(NO_3_)_2_), copper nitrate trihydrate (99%, Cu(NO_3_)_2_·3H2O), zinc nitrate hexahydrate (98%, Zn(NO_3_)_2_·6H_2_O), and cadmium nitrate tetrahydrate (98%, Cd(NO_3_)_2_·4H_2_O) were obtained from Daejung Chemical (Busan, Republic of Korea).

### 2.2. Immobilization of Spirulina platensis in Sodium Alginate

*Spirulina platensis* immobilized in sodium alginate was synthesized employing the following procedure. To minimize the salt content, *Spirulina platensis* powder was washed five times with distilled water and later dried for a period of 24 h at 100 °C in an oven. *Spirulina platensis* (2%, *w*/*v*) and SA (2%, *w*/*v*) were separately dissolved in deionized water and stirred to achieve uniform viscosity. After homogenization, the two solutions were mixed at a ratio of 1:1 and stirred overnight. Once homogeneous, the mixed slurries were incorporated into a solution containing 4% (*w*/*v*) CaCl_2_ using a peristaltic pump at a speed of 5 mL/min to form a spherical hydrogel based the bonding of the alginate group with Ca ions. The immobilized *Spirulina platensis* in sodium alginate (S.P@Ca-SA) remained in the aqueous solution for 1 h and was then washed with distilled water. Blank alginate beads were prepared using the same procedure as the S.P@Ca-SA beads, except for the inclusion of *Spirulina platensis* solution.

### 2.3. Characterization of Spirulina platensis and S.P@Ca-SA

The thermogravimetric behavior of *Spirulina platensis* was evaluated using TGA (SDT Q600, TA Instruments, New Castle, DE, USA). The analysis was conducted at a heating rate of 10 °C/min under N_2_ and a temperature range of 30–1000 °C. The surface functional groups of *Spirulina platensis* and S.P@Ca-SA were verified using a Fourier-transform infrared (FTIR) spectrometer (Nicolet 6700, Thermo Scientific, Waltham, MA, USA). The morphology and elements of the samples were detected using field-emission scanning electron microscopy (FE-SEM, S4800, Hitachi, Hitachi, Japan) with EDS (ISIS310, Jeol, Japan). The chemical bonding and elements were evaluated using X-ray photoelectron spectroscopy (XPS, Thermo Scientific K-Alpha spectrometer) with an X-ray source of Al Kα radiation. N_2_ adsorption-desorption isotherm evaluation was conducted using a surface area analyzer (BELSORP-max, BEL Japan Inc., Osaka, Japan). The zeta potentials under diverse pH conditions were characterized by zeta potential measurements using a Zetasizer Nano Analyzer (ZS 90, Malvern, UK). 

### 2.4. Batch Experiments

To investigate the initial adsorption of *Spirulina platensis*, divalent metal solutions of Pb(II), Cu(II), Zn(II), and Cd(II) at a concentration of 100 mg/L were prepared, and adsorption experiments were conducted (adsorbent dose = 1 g/L, 24 h). The effects of S.P@Ca-SA dosage, kinetics, equilibrium isotherm, initial solution pH, and recyclability were examined. Duplicate tests were executed to ensure data quality, and the mean values were calculated with standard deviations less than 5%. The effluent concentration in the filtrate was estimated using ICP-OES (Perkin Elmer Optima Model 5300DV, USA).

The effects of initial solution pH on Pb(II) removal were assessed at pH levels ranging from 1 to 7. The solution pH was adjusted using 0.1 M NaOH and 0.1 M HCl solutions and was measured using a pH probe (9107BN, Thermo Scientific, Waltham, MA, USA). Kinetic experiments (adsorbent dose = 1, 5, and 10 g/L; initial Pb(II) concentration = 100 mg/L, pH 5.1) were conducted at reaction times ranging from 10 min to 24 h. Equilibrium isotherm experiments (adsorbent dose = 1, 5, and 10 g/L; reaction time = 3 h) were conducted at initial Pb(II) concentrations ranging from 10 to 100 mg/L. The regeneration of S.P@Ca-SA is imperative for augmenting the cost-effectiveness of the adsorption process. Recyclability experiments were performed for five cycles of adsorption–desorption. The adsorption experiment was performed using S.P@Ca-SA (adsorbent dose = 1 g/L) and Pb(II) (100 mg/L) for 6 h. For each cycle, the Pb(II)-adsorbed S.P@Ca-SA was reacted for 1 h with 0.1M NaOH solution.

Pb(II) removal capacity (*qe*, mg/g) was calculated with the following equation:

(1) $$q_{e}=\frac{C_{i}-C_{f}}{C_{a}}$$

where *C_i_* is the Pb(II) concentration in the aqueous phase before the reaction (mg/L), *C_f_* is the Pb(II) concentration in the aqueous phase post-reaction (mg/L), and *C_a_* is the S.P@Ca-SA dose (g/L).

Kinetic adsorption data were analyzed using the following nonlinear forms of the pseudo-first-order (Equation (2)) and pseudo-second order (Equation (3)) models:

(2) $$q_{t}=q_{e}\left[1-\exp(-k_{1}t)\right]$$

(3) $$q_{t}=\frac{k_{2}q_{e}^{2}t}{1+k_{2}q_{e}t}$$

where *q_t_* is the amount of adsorbed Pb(II) per unit mass of S.P@Ca-SA (g/L) at time *t* (mg/g), and *q_e_* is the amount of adsorbed Pb(II) per unit mass of S.P@Ca-SA (g/L) at equilibrium (mg/g). *k*_1_ is the pseudo-first-order rate constant (1/h), and *k*_2_ is the pseudo-second-order rate constant (g/mg/h).

The equilibrium sorption data were analyzed using the following nonlinear forms of Langmuir (Equation (4)) and Freundlich (Equation (5)) isotherm models:

(4) $$q_{e}=\frac{Q_{m}K_{L}C_{e}}{1+K_{L}C_{e}}$$

(5) $$q_{e}=K_{F}C_{e}^{n}$$

where *Q_m_* is the maximum mass of adsorbed Pb(II) per unit mass of adsorbent (adsorption capacity, mg/g), and *C_e_* is the concentration of Pb(II) in the aqueous solution at equilibrium (mg/L). *K_L_* is the Langmuir constant connected to the binding energy (L/mg), *K_F_* is the Freundlich distribution coefficient (L/g), and 1/n is the Freundlich constant.

The following equations for the determination of coefficient (*R*^2^), chi-square coefficient (*χ*^2^), and sum of the squared error (SSE) were used to analyze the adsorption data and confirm their fit to the model:

(6) $$R^{2}=\frac{\sum_{i=1}^{m}{\left(y_{c}-\bar{y_{e}}\right)}_{i}^{2}}{\sum_{i=1}^{m}{\left(y_{c}-\bar{y_{e}}\right)}_{i}^{2}+\sum_{i=1}^{m}{\left(y_{c}-y_{e}\right)}_{i}^{2}}$$

(7) $$\chi^{2}=\sum_{i=1}^{m}{\left[\frac{{\left(y_{e}-y_{c}\right)}^{2}}{y_{c}}\right]}_{i}$$

(8) $$\mathrm{SSE}=\sum_{i=1}^{n}{\left(y_{e}-y_{c}\right)}^{2}$$

where $y_{c}$ is the removal capacity calculated from the model, $y_{e}$ is the removal capacity measured in the experiment, and $\bar{y_{e}}$ is the average measured removal capacity.

## 3. Results and Discussion

### 3.1. Characterization of Spirulina platensis

**Figure 1:**
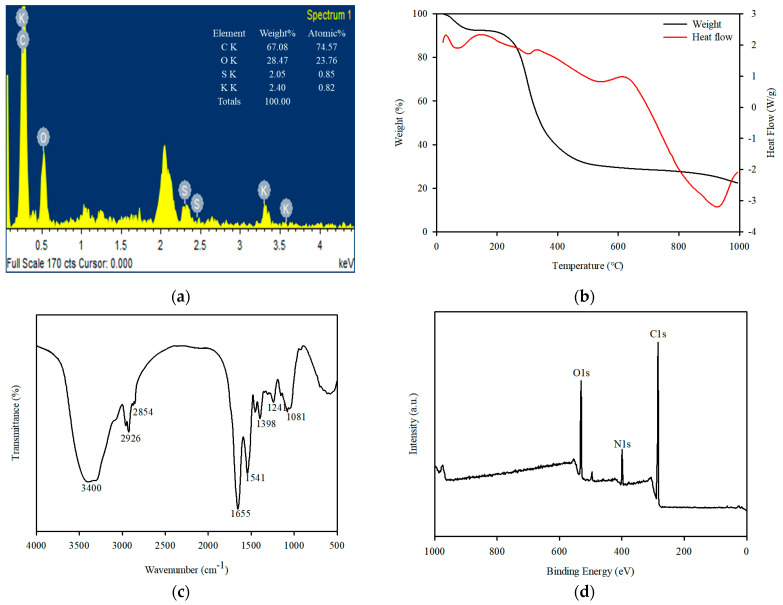
Characteristics of *Spirulina platensis*: (**a**) EDS pattern (inset = chemical composition), (**b**) TGA, (**c**) FTIR spectrum, and (**d**) XPS survey spectra.

An EDS investigation was performed to identify the elemental characteristics of *Spirulina platensis*. Accordingly, *Spirulina platensis* was primarily composed of C (74.5 wt%), O (23.7 wt%), S (0.85 wt%), and K (0.82 wt%) (Figure 1a). Carbon was the key element, and some trace chemical elements were adsorbed on the surface, indicating that they were derived from the growing media. The TG and DSC curves of *Spirulina platensis* at a heating rate of 10 °C/min under an inert N_2_ atmosphere are represented in Figure 1b. *Spirulina platensis* had a total mass loss of 78.3%, and the highest mass loss (68.7%) occurred at 500 °C, which corresponds to the loss of moisture, and cellulose, lipid, and protein decomposition [35,36].

**Figure 2:**
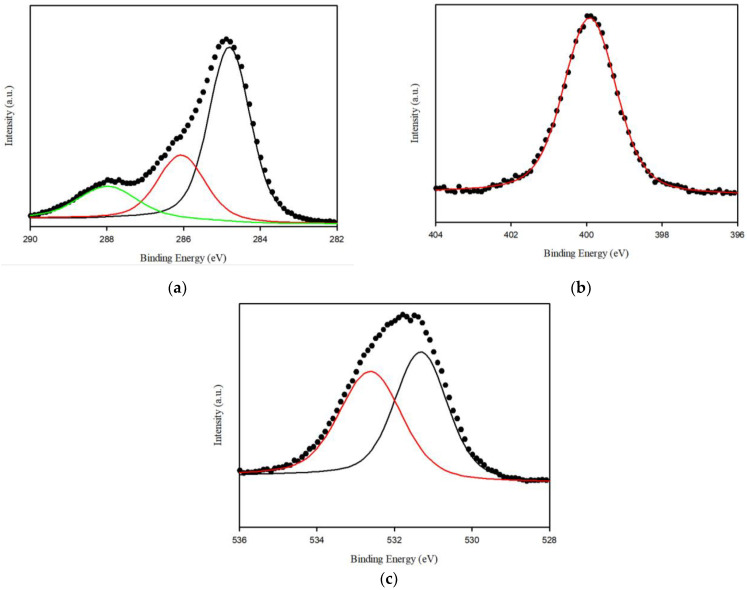
High resolution X-ray photoelectron spectroscopy (XPS) spectra for the surface of *Spirulina platensis*: (**a**) C 1 s (**b**) N 1 s and (**c**) O 1 s.

FTIR and XPS analyses were performed to verify the functional groups in *Spirulina platensis*. The FTIR spectrum of *Spirulina platensis* is presented in Figure 1c, demonstrating the presence of abundant functional groups on its surface. The FTIR spectrum indicated the presence of N-H and O-H (3400 cm^−1^), aliphatic C-H (3000–2900 cm^−1^), C=O (amide I, 1655 cm^−1^), and C-H stretching (1450 cm^−1^) accompanied by N-H binding (amide II, 1541 cm^−1^). A characteristic band was observed at approximately 1400–1000 cm^−1^ which was associated with S=O, C=C, C-O, and C-OH vibrations [37,38]. An evaluation of the XPS spectra was also performed to investigate the chemical composition of the surface of *Spirulina platensis*. The peaks at 284.4, 399.2, and 531.2 eV were assigned to C1s (73.6%), O1s (8.2%), and N1s (17.9%), respectively. In a high-resolution scan of N1s, the peak at 399.9 eV was attributed to protein-N (Figure 2) [39].

**Figure 3:**
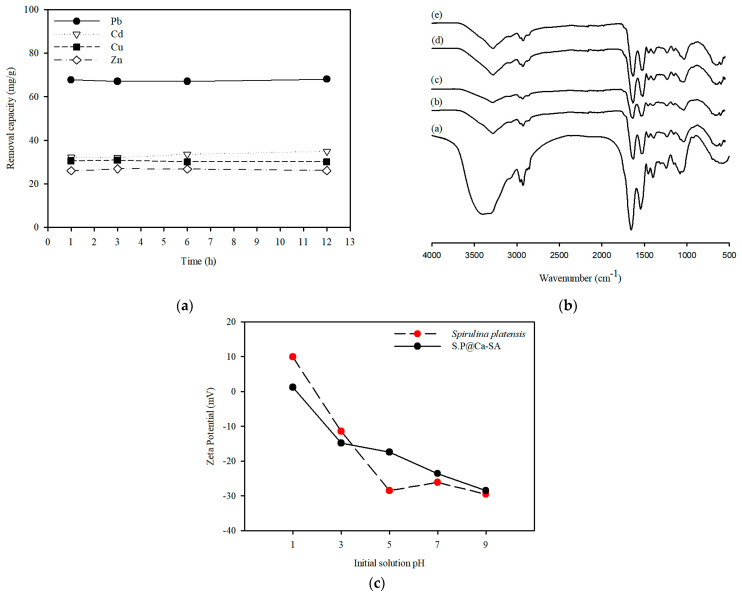
(**a**) Effect of contact time on adsorption capacity of Pb(Ⅱ), Cu(Ⅱ), Zn(Ⅱ), and Cd(Ⅱ) ions. (**b**) Fourier-transform infrared spectroscopy of *Spirulina platensis* before (a) and after adsorption (b–e) (Note: *Spirulina platensis* after adsorption using Pb(b), Cd(c), Cu(d), and Zn(e), initial concentration of 100 mg/L, respectively). (**c**) Zeta potential as a function of pH for *Spirulina platensis* and S.P@Ca-SA.

A preliminary adsorption study was conducted to explore the performance of *Spirulina platensis* dry biomass in heavy metal adsorption. The sorption properties of *Spirulina platensis* were assessed for Pb(II), Cu(II), Zn(II), and Cd(II) elimination with a 1.0 g/L adsorbent dose and an initial metal concentration of 100 mg/L. This was done to evaluate the heavy metal removal capacity. The capacity of *Spirulina platensis* for removal of metal cations was ranked as follows: Pb (68.0 mg/g) > Cd (34.8 mg/g) > Cu (30.0 mg/g) > Zn (26.1 mg/g) (as indicated in Figure 3a). The Pb removal capacities of various species of microalgae biomass reported in the literature are presented in Table 1. The adsorption capacity of heavy metals can be attributed to the plentiful functional groups, including amine, carboxyl, and hydroxyl groups, on the surface (Figure 3b). After *Spirulina platensis* adsorbed heavy metals, a decline in the OH stretching band intensity was confirmed. However, the metal cation removal by FTIR analysis indicated that there was no significant difference in the functional groups involved in the sorption process. Our results were similar to those of Ferreira et al. [40], who observed metal cation removal in *Spirulina platensis* using FTIR analysis. These studies have demonstrated that the functional groups involved in Pb(II) adsorption are the hydroxyl groups. Moreover, *Spirulina platensis* had an isoelectric point at pH 2.85 (Figure 3c). Thus, cationic metals can adsorb onto the negatively charged surface of *Spirulina platensis* through electrostatic interactions.

### 3.2. Characterization of SP@Ca-SA

**Figure 4:**
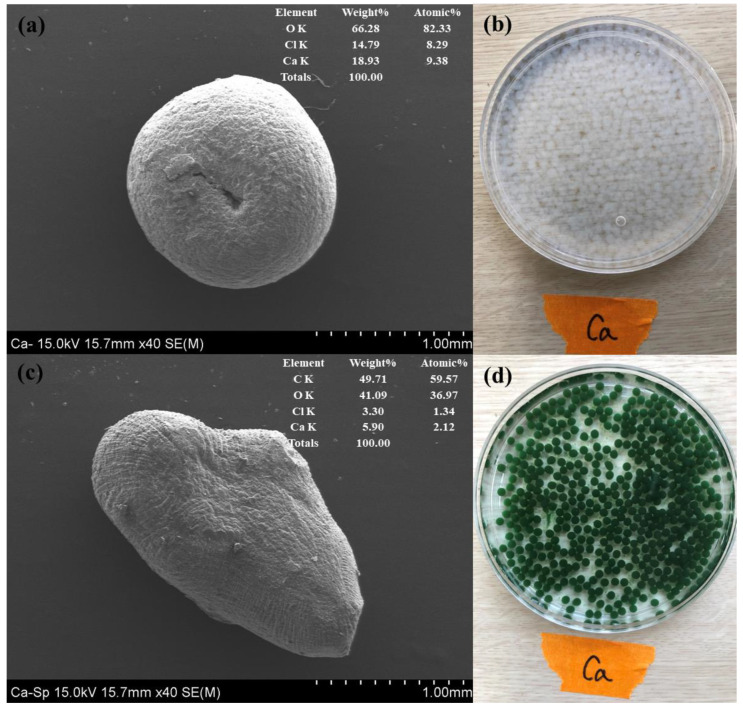
SEM-EDS images of Ca-SA (**a**) and S.P@Ca-SA (**c**) and digital images of Ca-SA (**b**) and S.P@Ca-SA (**d**).

Digital imaging established that calcium alginate was colorless, whereas S.P@Ca-SA was green. The morphology of S.P@Ca-SA was revealed using SEM, as depicted in Figure 4. Calcium alginate is spherically shaped and has a denser surface structure, whereas S.P@Ca-SA has an irregular surface and a more porous structure. The EDS of S.P@Ca-SA divulged the presence of C (59.5 wt%), O (36.9 wt%), Ca (2.12 wt%), and Cl (1.34 wt%). Post crosslinking of sodium alginate with calcium chloride, Ca and Cl were observed, indicating that alginate exists as a residue after crosslinking. The BET surface area of S.P@Ca-SA was determined to be 159.5 m^2^/g, which was higher than that of calcium alginate. Fila et al. (2022) reported that calcium alginate had a specific surface area of <5 m^2^/g [41]. It could be seen that S.P@Ca-SA had a more porous structure, implying the alterations in a greater number of sorption sites.

**Figure 5:**
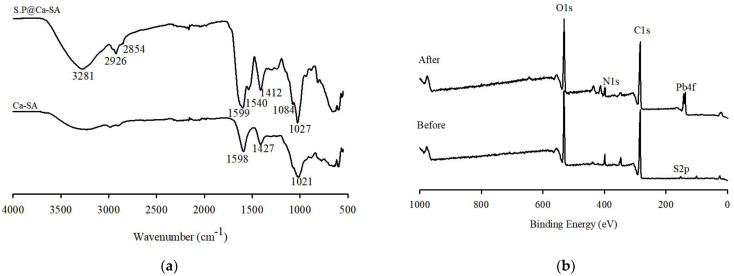
(**a**) FTIR of Ca-SA and S.P@Ca-SA. (**b**) XPS spectra of S.P@Ca-SA before and after adsorption.

The spectra of calcium alginate (Ca-SA) and S.P@Ca-SA were compared to identify the functional groups. The FTIR spectrum of S.P@Ca-SA is presented in Figure 5a, indicating that *Spirulina platensis* was successfully immobilized on the alginate matrix. The functional groups characterized by calcium-crosslinked alginate hydrogels indicated the presence of stretching vibrations of the O-H groups (about 3320 cm^−1^) and the C-O asymmetric (1598 cm^−1^) and C-O symmetric (1427 cm^−1^) vibrations in the COO groups [42,43]. This result indicates that Ca in the CaCl_2_ solution intercalates through an ion-exchange reaction with Na in the sodium alginate. S.P@Ca-SA depicted a similar formation of functional groups, and novel characteristic peaks appearing for S.P@Ca-SA compared with calcium alginate. Among these peaks, those at 2854 and 2926 cm^−1^ were assigned to CH_2_ symmetric and CH_2_ asymmetric stretching, respectively, and those at 1541 and 1412 cm^−1^ were assigned to the N-H bending vibration. Furthermore, peaks at 1084 and 1027 cm^−1^ were identified as C-N and C-O stretching vibrations, respectively.

**Figure 6:**
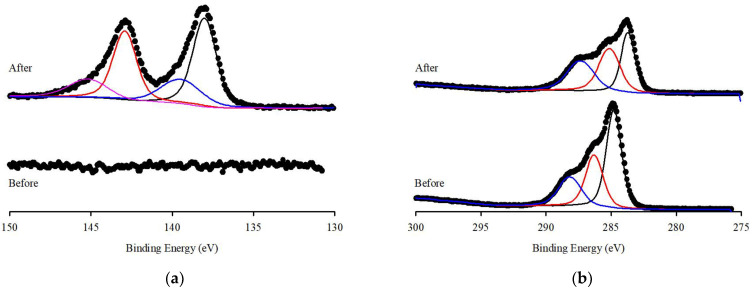

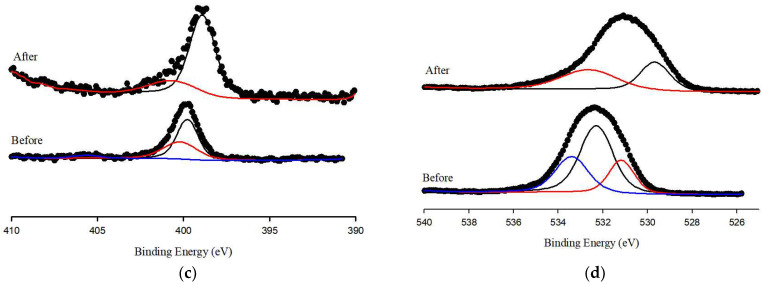
XPS spectrum of (**a**) Pb 4f, (**b**) C 1s, (**c**) N 1s and (**d**) O 1s of S.P@Ca-SA before and after reaction.

XPS spectra were evaluated to detect the functional groups present before and after the adsorption of Pb(II) ions onto S.P@Ca-SA. The wide scans of S.P@Ca-SA are depicted in Figure 5b; the characteristic peaks pre-adsorption at binding energies of 284.8, 399.1, and 531.4 eV are attributed to C1s, N1s, and O1s, respectively. Post adsorption of Pb(II), a new characteristic peak appeared at a binding energy of 138.3 eV, which was attributed to Pb 4f [44,45,46]. Compared with Pb(NO_3_)_2_, a lower binding energy of 139.6 eV for Pb 4f was observed, which was associated with the affinity of Pb(II) and S.P@Ca-SA [47]. The high-resolution spectra of N1s of S.P@Ca-SA are shown in Figure 6. The peaks at 399 and 400 eV were attributed to NH_2_ and NH_3_, implicating that *Spirulina platensis* successfully immobilized into alginate. After Pb(II) adsorption, the NH_2_ peak shifted to lower binding energies, indicating complexation between the NH_2_ groups and Pb(II). The peaks at 284.8, 286.4, and 288.0 eV in the C1s spectrum of S.P@Ca-SA were assigned to C-C, C-N, and O-C=O, respectively. The O1s spectrum exhibited peaks at 531.8, 532.5, and 533.4 eV, which were ascribed to C=O, C-O, and O-C=O, respectively. Compared with the XPS spectrum before Pb(II) adsorption, the spectra of both C1s and O1s demonstrated obvious changes after adsorption.

### 3.3. Adsorption Experiments

#### 3.3.1. Effect of Initial pH on the Adsorption

**Figure 7:**
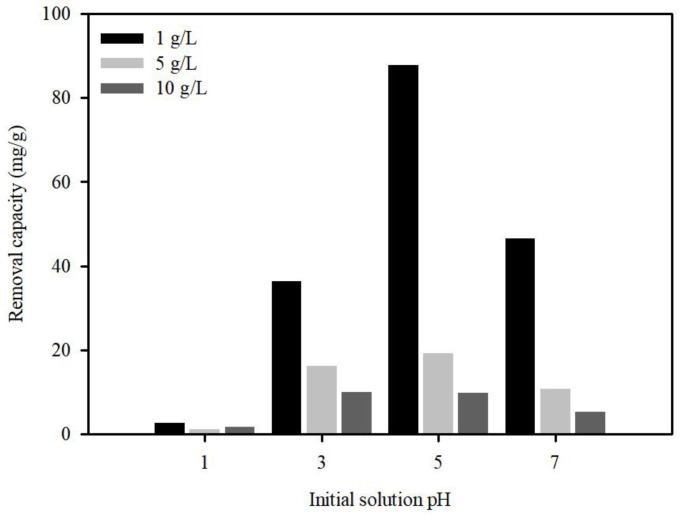
Adsorption of Pb(II) on S.P@Ca-SA as affected by aqueous pH.

The initial pH of Pb(II) plays a critical role in adsorption due to Pb speciation in the solution, surface charge, and competition for available sorption sites. To investigate the effect of pH, pH levels varying from 1 to 7 were established by adjusting the initial solution pH using 0.1 M NaOH or HCl. The major forms of Pb(II) present in aqueous solutions are dependent on pH [48,49]. Therefore, this initial pH range was selected to avoid precipitation of Pb(OH)_2_. The effects of initial pH on Pb(II) removal by S.P@Ca-SA are shown in Figure 7. As expected, Pb(II) adsorption onto S.P@Ca-SA depended on the pH value, and the removal capacity was augmented with increasing pH. Increasing doses of S.P@Ca-SA at pH 5 reduced the removal capacity from 87.9 to 9.98 mg/g. Under highly acidic conditions at pH 1, Pb(II) sorption declined sharply, which could be ascribed to the competition between Pb(II) and H^+^ ions for sorption sites [50]. This suggests the contribution of the effects of protonation or deprotonation of functional groups on the S.P@Ca-SA surface. Moreover, the zeta potential charge (pH_ZPC_) of S.P@Ca-SA was found to be 1.17, which was related to the surface state of S.P@Ca-SA. When the solution pH was above pH_ZPC_, the adsorption capacity of S.P@Ca-SA indicated that the influence of ionic groups, such as carboxylate, was more conducive to the adsorption efficiency of Pb(II).

#### 3.3.2. Equilibrium Isotherms and Kinetic Model Analyses

**Figure 8:**
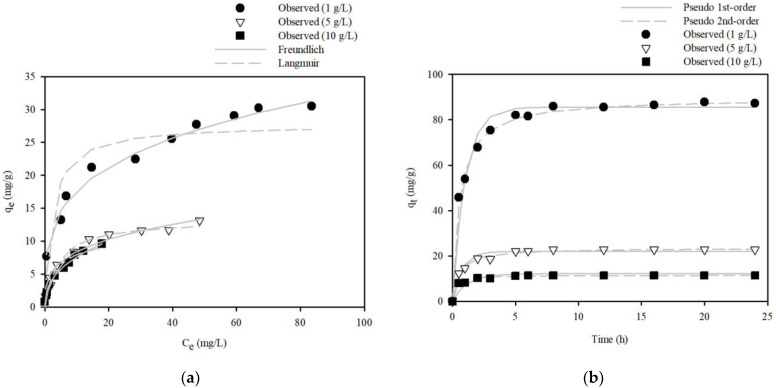
(**a**) Adsorption isotherm of Pb(Ⅱ) onto the S.P@Ca-SA, and (**b**) Pb(Ⅱ) adsorption kinetics fitted by pseudo-first-order and pseudo-second-order models onto the S.P@Ca-SA.

The effect of the dose of S.P@Ca-SA on Pb(II) removal capacity was studied using an initial concentration ranging from 10 to 100 mg/L, with shaking for 180 min, and the results are represented in Figure 8a. With increasing doses of S.P@Ca-SA, the removal capacity of S.P@Ca-SA diminished (amount of Pb(II) adsorbed per unit mass), whereas the removal efficiency of Pb(II) was augmented. When the initial concentration was 100 mg/L, as the adsorbent dose increased from 1 to 10 g/L, the maximum removal capacity reduced from 30.5 to 9.60 mg/g. Additionally, the removal capacity of S.P@Ca-SA at 1 g/L dosage increased from 7.68 to 30.5 mg/g for Pb(II) as the initial concentration escalated. Our results demonstrate that the number of sorption sites available for Pb(II) was augmented, resulting in an enhanced removal capacity and availability for adsorption at higher concentrations. To analyze the type of Pb(II) adsorption, equilibrium isotherm models, including the Langmuir and Freundlich models, were adopted for data fitting. The equilibrium model parameters are listed in Table 2. The fitting results, as compared using the two equilibrium isotherm models, revealed that the Freundlich model is most suitable for describing the equilibrium data with parameter values of K_F_ = 9.48–2.99 L/g and 1/n = 0.27–0.40.

The adsorption kinetics were examined with diverse doses of S.P@Ca-SA for Pb(II) removal at the initial concentration (C_0_ = 100 mg/L), and the results are depicted in Figure 8b. S.P@Ca-SA attained equilibrium after 6 h of contact with the Pb(II) solution. To evaluate the kinetic data in the study, kinetic sorption models, including pseudo-first-order and -second-order models, were fitted, and the error functions for R^2^, χ^2^, and SAE were employed to evaluate the experimental data. The calculated kinetic parameters and correlation coefficients are enlisted in Table 3. R^2^, χ^2^, and SSE values of the pseudo-second-order model were higher than those of the first-order model. Therefore, it is concluded that the pseudo-second-order model is more suitable for describing the kinetic data with parameter values of q_e_ = 89.6–11.6 mg/g and k_2_ = 0.02–0.39 g/mg/h. The Pb removal capacities of various biomass-based adsorbents reported in the literature are presented in Table 4.

#### 3.3.3. Recyclability of SP@Ca-SA

**Figure 9:**
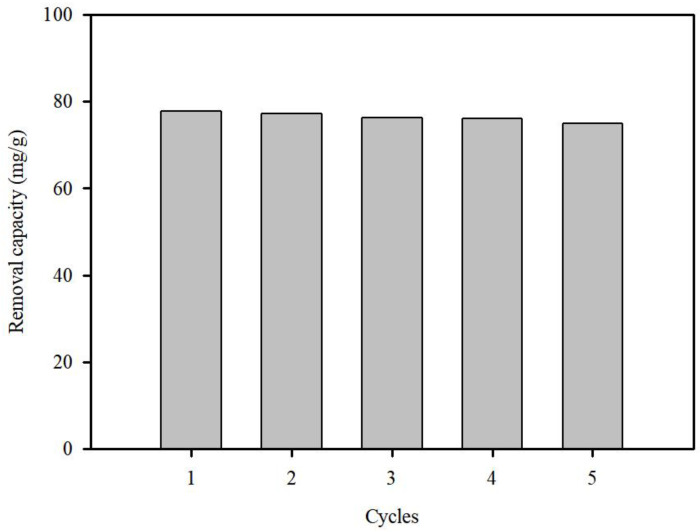
Regeneration studies of S.P@Ca-SA for Pb(II) over five cycles.

The regeneration ability of S.P@Ca-SA was studied using Pb(II) adsorption–desorption experiments. Herein, 0.1 NaOH was selected to regenerate the adsorbent. Post adsorption, equilibrium was attained (77.7 mg/g), and the adsorbed Pb(II) was desorbed at 87% within 60 min. A regeneration experiment with S.P@Ca-SA was conducted, as depicted in Figure 9. The Pb(II) adsorption capacity of S.P@Ca-SA was maintained at the same level even after five regeneration cycles, which indicates that S.P@Ca-SA can be reused with good durability.

### 3.4. Adsorption Mechanisms

**Figure 10:**
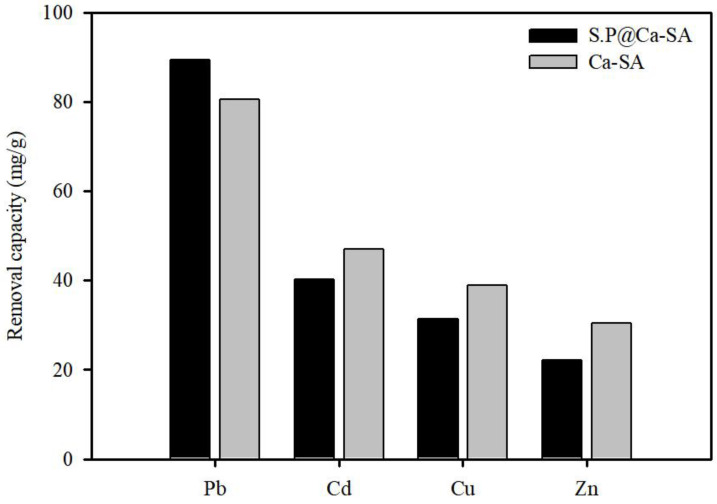
Removal capacity of Ca-SA and S.P@Ca-SA for Pb(Ⅱ), Cu(Ⅱ), Zn(Ⅱ), and Cd(Ⅱ) ions.

Ca-SA and S.P@Ca-SA were compared to evaluate the effects of *Spirulina platensis* immobilization. Batch experiments were performed for divalent metal ion solutions, Pb, Cu, Zn, and Cd, at 100 mg/L concentration (Figure 10). Ca-SA had a relatively lower affinity for Pb(II) than S.P@Ca-SA. This implies that the enhanced adsorption capacity and selectivity were due to the introduction of alginate into the N-containing functional groups. Under the given experimental conditions, the Pb(II) removal capacity was the highest, while the relative removal efficiencies of several divalent metal ions tended to be low. A similar observation was reported by Zhang et al. (2020), who established that this tendency could be attributed to the stability constant of the associated metal acetate and hydroxide [57]. Other researchers have also reported that the contribution of competitive adsorption is influenced by electronegativity and ionic radius [58,59].

From the above FTIR and XPS results, Ca-SA had typical O-containing functional groups, including -OH and -COOH on the surface, indicating that Pb(II) adsorption was primarily electrostatic. Meanwhile, S.P@Ca-SA had N-containing functional groups, suggesting that the amino groups had an impact on Pb(II) removal. The Pb(II) adsorption capacity of S.P@Ca-SA was ascribed to complexation between the amino groups and Pb(II). This signifies that a stable coordination complex was formed by chelation between the amino groups and Pb(II). Compared with the XPS spectrum before Pb(II) adsorption, the change in the binding energies indicated that the N-containing functional groups (N1s) may participate in the removal process. Moreover, positively charged Pb(II) can adsorb onto the negatively charged carboxyl and hydroxyl functional groups present in S.P@Ca-SA through electrostatic interactions.

## 4. Conclusions

*Spirulina platensis* was incorporated using an in situ crosslinking process to the alginate matrix, which resulted in an increased Pb(II) adsorption capacity. The FTIR and XPS spectral patterns of S.P@Ca-SA verified that functional groups, including amine, carboxyl, and hydroxyl groups, were associated with the alginate matrix. For *Spirulina platensis*, Ca-SA, and S.P@Ca-SA, the maximum adsorbed amount of Pb(II) reached 68.0 mg/g, 80.5 mg/g, and 89.4 mg/g, respectively. Compared with *Spirulina platensis* and blank alginate beads, S.P@Ca-SA exhibited an enhanced Pb(II) adsorption efficiency. The improvement in Pb(II) adsorption with the immobilization of *Spirulina platensis* may be associated with functional groups and the availability of more adsorption sites. Batch experiments demonstrated that S.P@Ca-SA could effectively remove Pb(II) at pH 5, reaching equilibrium within 6 h, and the maximum Pb(II) sorption capacity of S.P@Ca-SA was 87.9 mg/g. Our results indicated that S.P@Ca-SA fits well with the pseudo-second-order and Freundlich models. The adsorption results demonstrated that complexation and electrostatic attraction could be the mechanism underlying Pb(II) adsorption. Overall, the regeneration efficiency of S.P@Ca-SA was excellent for up to five regeneration cycles. S.P@Ca-SA could be a promising adsorbent for the removal of Pb(II) from aqueous solutions.

## Figures and Tables

**Table 1:** Pb removal capacities of various species of microalgae biomass reported in the literature [33].

Species	Initial Metal Concentration (mg/L)	Type of Wastewater	Removal Capacity (mg/g)
*Chaetoceros* sp.	20	Synthetic	46.94
*Chlorella* sp.	50	Synthetic	20.95
*Spirogyra*	500	Real	94.34
*Cyanothece* sp.	500	Synthetic	28.57
*Phormidium* sp.	10	Synthetic	5.15
*Rhizoclonium hookeri*	1000	Synthetic	81.7
*Spirulina platensis*	100	Synthetic	68.0

**Table 2:** Equilibrium isotherm model parameters obtained from model fitting to experimental data.

**Adsorbent Dose** **(g/L)**	**Freundlich Model**				
	**K_F_** **(L/g)**	**1/n**	**R^2^**	$\chi^{2}$	**SSE**
1	9.48	0.27	0.98	0.49	8.42
5	4.32	0.29	0.96	1.37	6.33
10	2.99	0.40	0.99	0.90	2.51
**Adsorbent Dose** **(g/L)**	**Langmuir Model**				
	**Q_m_** **(mg/g)**	**K_L_** **(L/mg)**	**R^2^**	** $\chi^{2}$ **	**SSE**
1	27.7	0.43	0.81	5.64	29.3
5	13.2	0.25	0.98	1.83	4.61
10	10.8	0.28	0.98	47.4	4.93

**Table 3:** Kinetic model parameters obtained from model fitting to experimental data.

**Adsorbent Dose** **(g/L)**	**Pseudo First-Order Model**				
	**q_e_** **(mg/g)**	**k_1_** **(1/h)**	**R^2^**	** $\chi^{2}$ **	**SSE**
1	75.5	1.03	0.92	3.48	37.2
5	21.4	0.87	0.90	1.79	11.7
10	11.4	1.25	0.84	1.53	4.07
**Adsorbent Dose** **(g/L)**	**Pseudo Second-Order Model**				
	**q_e_** **(mg/g)**	**k_2_** **(g/mg/h)**	**R^2^**	** $\chi^{2}$ **	**SSE**
1	80.3	0.01	0.99	0.56	13.3
5	22.8	0.06	0.97	0.36	5.24
10	11.6	0.39	0.89	0.25	2.88

**Table 4:** Pb removal capacities of various biomass-based adsorbents reported in the literature.

Adsorbent	Removal Capacity (mg/g)	Reference
Lignin microspheres	33.9	[51]
Lignin-chitin hybrid material	75.7	[52]
Aminated epoxy-lignin	72.4	[53]
Bi-functionlized lignin	53.8	[54]
1, 2, 4-triazole modified lignin	42	[55]
Chitosan-vanillin derivatives of chelating polymers	23.3	[56]
S.P@Ca-SA	87.9	This study

## Data Availability

All data generated or analyzed during this study are included in this published article.

## References

[1] Ye Z., Yin X., Chen L., He X., Lin Z., Liu C., Ning S., Wang X., Wei Y. (2019). An integrated process for removal and recovery of Cr(VI) from electroplating wastewater by ion exchange and reduction–precipitation based on a silica-supported pyridine resin. J. Clean. Prod..

[2] Wen J., Jiang T., Wang J., Gao H., Lu L. (2019). An efficient utilization of high chromium vanadium slag: Extraction of vanadium based on manganese carbonate roasting and detoxification processing of chromium-containing tailings. J. Hazard. Mater..

[3] Król A., Mizerna K., Bożym M. (2019). An assessment of pH-dependent release and mobility of heavy metals from metallurgical slag. J. Hazard. Mater..

[4] Collin M.S., Venkatraman S.K., Vijayakumar N., Kanimozhi V., Arbaaz S.M., Stacey R.G.S., Anusha J., Choudhary R., Lvov V., Tovar G.I. (2022). Bioaccumulation of lead (Pb) and its effects on human: A review. J. Hazard. Mater. Adv..

[5] Bai C., Wang L., Zhu Z. (2020). Adsorption of Cr(III) and Pb(II) by graphene oxide/alginate hydrogel membrane: Characterization, adsorption kinetics, isotherm and thermodynamics studies. Int. J. Biol. Macromol..

[6] Chiew C.S.C., Yeoh H.K., Pasbakhsh P., Krishnaiah K., Poh P.E., Tey B.T., Chan E.S. (2016). Halloysite/alginate nanocomposite beads: Kinetics, equilibrium and mechanism for lead adsorption. Appl. Clay Sci..

[7] Wu D., Hu L., Wang Y., Wei Q., Yan L., Yan T., Li Y., Du B. (2018). EDTA modified β-cyclodextrin/chitosan for rapid removal of Pb(II) and acid red from aqueous solution. J. Colloid Interface Sci..

[8] Jiang L., Li Y., Pei H. (2021). Algal–bacterial consortia for bioproduct generation and wastewater treatment. Renew. Sustain. Energy Rev..

[9] Qu P., Li Y., Huang H., Chen J., Yu Z., Huang J., Wang H., Gao B. (2020). Urea formaldehyde modified alginate beads with improved stability and enhanced removal of Pb^2+^, Cd^2+^, and Cu^2+^. J. Hazard. Mater..

[10] Long Y., Yu G., Dong L., Xu Y., Lin H., Deng Y., You X., Yang L., Liao B.-Q. (2021). Synergistic fouling behaviors and mechanisms of calcium ions and polyaluminum chloride associated with alginate solution in coagulation-ultrafiltration (UF) process. Water Res..

[11] Ahmad Z., Li Y., Huang C., Gou X., Fan Y., Chen J. (2021). Underwater suspended bifunctionalized polyethyleneimine-based sponge for selective removal of anionic pollutants from aqueous solution. J. Hazard. Mater..

[12] Mo Y., Vincent T., Faur C., Guibal E. (2020). Se(VI) sorption from aqueous solution using alginate/polyethylenimine membranes: Sorption performance and mechanism. Int. J. Biol. Macromol..

[13] Xu X., Wang B., Tang H., Jin Z., Mao Y., Huang T. (2020). Removal of phosphate from wastewater by modified bentonite entrapped in Ca-alginate beads. J. Environ. Manag..

[14] Pap S., Bezanovic V., Radonic J., Babic A., Saric S., Adamovic D., Turk Sekulic M. (2018). Synthesis of highly-efficient functionalized biochars from fruit industry waste biomass for the removal of chromium and lead. J. Mol. Liq..

[15] Li Z., Wang L., Wu J., Xu Y., Wang F., Tang X., Xu J., Ok Y.S., Meng J., Liu X. (2020). Zeolite-supported nanoscale zero-valent iron for immobilization of cadmium, lead, and arsenic in farmland soils: Encapsulation mechanisms and indigenous microbial responses. Environ. Pollut..

[16] Acosta R., Fierro V., Martinez de Yuso A., Nabarlatz D., Celzard A. (2016). Tetracycline adsorption onto activated carbons produced by KOH activation of tyre pyrolysis char. Chemosphere.

[17] Law X.N., Cheah W.Y., Chew K.W., Ibrahim M.F., Park Y.-K., Ho S.-H., Show P.L. (2022). Microalgal-based biochar in wastewater remediation: Its synthesis, characterization and applications. Environ. Res..

[18] Bhadra B.N., Jhung S.H. (2020). Adsorptive removal of nitrogenous compounds from microalgae-derived bio-oil using metal-organic frameworks with an amino group. Chem. Eng. J..

[19] Xiong C., Xue C., Huang L., Hu P., Fan P., Wang S., Zhou X., Yang Z., Wang Y., Ji H. (2021). Enhanced selective removal of Pb(II) by modification low-cost bio-sorbent: Experiment and theoretical calculations. J. Clean. Prod..

[20] Jaiswal K.K., Kumar V., Verma R., Verma M., Kumar A., Vlaskin M.S., Nanda M., Kim H. (2021). Graphitic bio-char and bio-oil synthesis via hydrothermal carbonization-co-liquefaction of microalgae biomass (oiled/de-oiled) and multiple heavy metals remediations. J. Hazard. Mater..

[21] Huang R., Huo G., Song S., Li Y., Xia L., Gaillard J.-F. (2019). Immobilization of mercury using high-phosphate culture-modified microalgae. Environ. Pollut..

[22] Sayadi M.H., Rashki O., Shahri E. (2019). Application of modified *Spirulina platensis* and *Chlorella vulgaris* powder on the adsorption of heavy metals from aqueous solutions. J. Environ. Chem. Eng..

[23] Rai A., Sirotiya V., Mourya M., Khan M.J., Ahirwar A., Sharma A.K., Kawatra R., Marchand J., Schoefs B., Varjani S. (2022). Sustainable treatment of dye wastewater by recycling microalgal and diatom biogenic materials: Biorefinery perspectives. Chemosphere.

[24] Leong Y.-K., Chang J.-S. (2020). Bioremediation of heavy metals using microalgae: Recent advances and mechanisms. Bioresour. Technol..

[25] Leng L., Wei L., Xiong Q., Xu S., Li W., Lv S., Lu Q., Wan L., Wen Z., Zhou W. (2020). Use of microalgae based technology for the removal of antibiotics from wastewater: A review. Chemosphere.

[26] Peres E.C., Cunha J.M., Dortzbacher G.F., Pavan F.A., Lima É.C., Foletto E.L., Dotto G.L. (2018). Treatment of leachates containing cobalt by adsorption on *Spirulina* sp. and activated charcoal. J. Environ. Chem. Eng..

[27] Nithya K., Sathish A., Pradeep K., Kiran Baalaji S. (2019). Algal biomass waste residues of *Spirulina platensis* for chromium adsorption and modeling studies. J. Environ. Chem. Eng..

[28] Pez Jaeschke D., Rocha Teixeira I., Ferreira Marczak L.D., Mercali G.D. (2021). Phycocyanin from *Spirulina*: A review of extraction methods and stability. Food Res. Int..

[29] Chakraborty I., Bhowmick G.D., Ghosh D., Dubey B.K., Pradhan D., Ghangrekar M.M. (2020). Novel low-cost activated algal biochar as a cathode catalyst for improving performance of microbial fuel cell. Sustain. Energy Technol. Assess..

[30] Cho D.-W., Yoon K., Kwon E.E., Biswas J.K., Song H. (2017). Fabrication of magnetic biochar as a treatment medium for As(V) via pyrolysis of FeCl_3_-pretreated spent coffee ground. Environ. Pollut..

[31] Liu Y., Xu J., Cao Z., Fu R., Zhou C., Wang Z., Xu X. (2020). Adsorption behavior and mechanism of Pb(II) and complex Cu(II) species by biowaste-derived char with amino functionalization. J. Colloid Interface Sci..

[32] Ma C., Zhao Y., Chen H., Liu Y., Huang R., Pan J. (2022). Biochars derived from by-products of microalgae pyrolysis for sorption of gaseous H_2_S. J. Environ. Chem. Eng..

[33] Nateras-Ramírez O., Martínez-Macias M.R., Sánchez-Machado D.I., López-Cervantes J., Aguilar-Ruiz R.J. (2022). An overview of microalgae for Cd^2+^ and Pb^2+^ biosorption from wastewater. Bioresour. Technol. Rep..

[34] Sun X., Huang H., Zhao D., Lin J., Gao P., Yao L. (2020). Adsorption of Pb^2+^ onto freeze-dried microalgae and environmental risk assessment. J. Environ. Manag..

[35] Binda G., Spanu D., Bettinetti R., Magagnin L., Pozzi A., Dossi C. (2020). Comprehensive comparison of microalgae-derived biochar from different feedstocks: A prospective study for future environmental applications. Algal Res..

[36] Akbarbaglu Z., Ayaseh A., Ghanbarzadeh B., Sarabandi K. (2022). Techno-functional, biological and structural properties of *Spirulina platensis* peptides from different proteases. Algal Res..

[37] Çelekli A., Yavuzatmaca M., Bozkurt H. (2010). An eco-friendly process: Predictive modelling of copper adsorption from aqueous solution on *Spirulina platensis*. J. Hazard. Mater..

[38] Choi Y.-K., Choi T.-R., Gurav R., Bhatia S.K., Park Y.-L., Kim H.J., Kan E., Yang Y.-H. (2020). Adsorption behavior of tetracycline onto *Spirulina* sp. (microalgae)-derived biochars produced at different temperatures. Sci. Total Environ..

[39] Guo D., Wang Y., Gao Y., Lyu Y., Lin Y., Pan Y., Zhu L., Zhu Y. (2022). Nitrogen migration in products during the microwave-assisted hydrothermal carbonization of *spirulina platensis*. Bioresour. Technol..

[40] Ferreira L.S., Rodrigues M.S., de Carvalho J.C.M., Lodi A., Finocchio E., Perego P., Converti A. (2011). Adsorption of Ni^2+^, Zn^2+^ and Pb^2+^ onto dry biomass of *Arthrospira (Spirulina) platensis* and *Chlorella vulgaris.* I. Single metal systems. Chem. Eng. J..

[41] Fila D., Hubicki Z., Kołodyńska D. (2022). Applicability of new sustainable and efficient alginate-based composites for critical raw materials recovery: General composites fabrication optimization and adsorption performance evaluation. Chem. Eng. J..

[42] Jiang X., Wang H., Wang Q., Hu E., Duan Y. (2020). Immobilizing amino-functionalized mesoporous silica into sodium alginate for efficiently removing low concentrations of uranium. J. Clean. Prod..

[43] Hu C., Lu W., Mata A., Nishinari K., Fang Y. (2021). Ions-induced gelation of alginate: Mechanisms and applications. Int. J. Biol. Macromol..

[44] Yang K., Lou Z., Fu R., Zhou J., Xu J., Baig S.A., Xu X. (2018). Multiwalled carbon nanotubes incorporated with or without amino groups for aqueous Pb(II) removal: Comparison and mechanism study. J. Mol. Liq..

[45] Luo Z., Chen H., Wu S., Yang C., Cheng J. (2019). Enhanced removal of bisphenol A from aqueous solution by aluminum-based MOF/sodium alginate-chitosan composite beads. Chemosphere.

[46] Liu Q., Wu H., Chen J., Guo B., Zhao X., Lin H., Li W., Zhao X., Lv S., Huang C. (2022). Adsorption mechanism of trace heavy metals on microplastics and simulating their effect on microalgae in river. Environ. Res..

[47] Gong D., Li B., Celi N., Cai J., Zhang D. (2021). Efficient Removal of Pb(II) from Aqueous Systems Using *Spirulina*-Based Biohybrid Magnetic Helical Microrobots. ACS Appl. Mater. Interfaces.

[48] Zhao Y., Zhang R., Liu H., Li M., Chen T., Chen D., Zou X., Frost R.L. (2019). Green preparation of magnetic biochar for the effective accumulation of Pb(II): Performance and mechanism. Chem. Eng. J..

[49] Esfandiar N., Suri R., McKenzie E.R. (2022). Competitive sorption of Cd, Cr, Cu, Ni, Pb and Zn from stormwater runoff by five low-cost sorbents; Effects of co-contaminants, humic acid, salinity and pH. J. Hazard. Mater..

[50] Tang S., Lin L., Wang X., Feng A., Yu A. (2020). Pb(II) uptake onto nylon microplastics: Interaction mechanism and adsorption performance. J. Hazard. Mater..

[51] Ge Y., Qin L., Li Z. (2016). Lignin microspheres: An effective and recyclable natural polymer-based adsorbent for lead ion removal. Mater. Des..

[52] Bartczak P., Klapiszewski Ł., Wysokowski M., Majchrzak I., Czernicka W., Piasecki A., Ehrlich H., Jesionowski T. (2017). Treatment of model solutions and wastewater containing selected hazardous metal ions using a chitin/lignin hybrid material as an effective sorbent. J. Environ. Manag..

[53] Liu X., Zhu H., Qin C., Zhou J., Zhao J.R., Wang S. (2013). Adsorption of heavy metal ion from aqueous single metal solution by aminated epoxy-lignin. BioResources.

[54] Ge Y., Li Z., Kong Y., Song Q., Wang K. (2014). Heavy metal ions retention by bi-functionalized lignin: Synthesis, applications, and adsorption mechanisms. J. Ind. Eng. Chem..

[55] Jin C., Zhang X., Xin J., Liu G., Wu G., Kong Z., Zhang J. (2017). Clickable synthesis of 1, 2, 4-triazole modified lignin-based adsorbent for the selective removal of Cd(II). ACS Sustain. Chem. Eng..

[56] Alakhras F., Al-Shahrani H., Al-Abbad E., Al-Rimawi F., Ouerfelli N. (2019). Removal of Pb(II) Metal Ions from Aqueous Solutions Using Chitosan-Vanillin Derivatives of Chelating Polymers. Pol. J. Environ. Stud..

[57] Zhang M., Yin Q., Ji X., Wang F., Gao X., Zhao M. (2020). High and fast adsorption of Cd(II) and Pb(II) ions from aqueous solutions by a waste biomass based hydrogel. Sci. Rep..

[58] Ma Y., Deng Z., Li Z., Lin Q., Wu Y., Dou W. (2021). Adsorption characteristics and mechanism for K_2_Ti_4_O_9_ whiskers removal of Pb(II), Cd(II), and Cu(II) cations in wastewater. J. Environ. Chem. Eng..

[59] Bo S., Luo J., An Q., Xiao Z., Wang H., Cai W., Zhai S., Li Z. (2020). Efficiently selective adsorption of Pb(II) with functionalized alginate-based adsorbent in batch/column systems: Mechanism and application simulation. J. Clean. Prod..

